# Generative semantic reconstruction for annotation-ready vegetation priors in spectrally heterogeneous imagery

**DOI:** 10.1016/j.mex.2026.103967

**Published:** 2026-05-20

**Authors:** Shubham Rana, Oliver Hensel, Abozar Nasirahmadi

**Affiliations:** a Department of Agricultural and Biosystems Engineering, University of Kassel, Witzenhausen, D-37213, Germany; b Department of Energy and Technology, Swedish University of Agricultural Sciences, Box 7032, Uppsala 75007, Sweden

**Keywords:** Generative semantic reconstruction, Annotation-ready vegetation priors, Prompt-constrained image reconstruction, Foundation model segmentation, Generative AI, Rangeland

## Abstract

Monitoring vegetation in arid rangelands is challenging because exposed soil, dry litter, shadows, and sparse canopy structure reduce the stability of standard segmentation workflows. This article presents a Google Gemini generative-AI-assisted methodology for producing annotation-ready vegetation priors from visually heterogeneous rangeland imagery. The workflow was applied to RGB images of indigenous forage species from Marsabit County, Kenya, where semantic reconstruction transformed noisy field scenes into structured representations for staged scene parsing, mask refinement, and final object-mask generation. Paired spectral analysis across 98 original and generated image pairs showed that reconstruction produced a measurable spectral-domain shift rather than simple background-variance reduction. Background coefficient of variation increased by 125.4 %, background Shannon entropy by 49.8 %, and target-vegetation Coefficient of Variation (CoV) by 11.4 %, indicating texture redistribution after reconstruction. Jeffries-Matusita separability changed only slightly, with a global shift of −0.8 %, suggesting that vegetation-background distinction remained close to saturation. The reconstruction-enhanced priors were used for automated annotation and evaluated using YOLOv8, YOLOv11, and RF-DETR. The protocol converts noisy, spectrally heterogeneous rangeland imagery into segmentation-ready training data for ecological computer vision, although broader geographic generalization requires validation on independent dryland datasets.

## Graphical abstract

**Figure:**
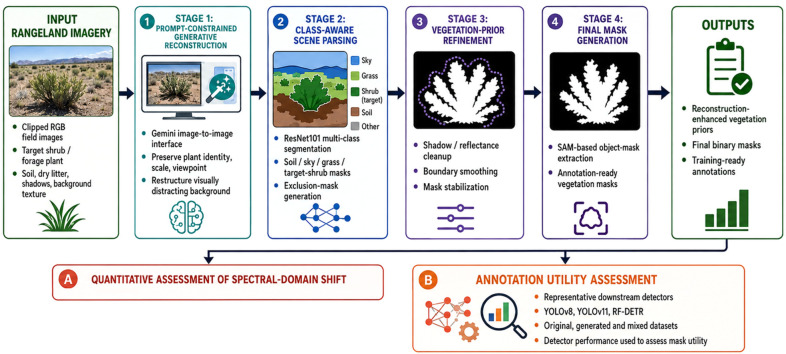

**Specifications table**

**Table:** 

**Subject area**	Computer Science
**More specific subject area**	Generative AI
**Name of your method**	Generative Semantic Reconstruction
**Name and reference of original method**	Not Applicable
**Resource availability**	Prompt templates, parameter settings, and sample data will be made available upon request

## Background

Rangeland ecosystems represent critical land-use systems that sustain ecological processes, biodiversity, and the livelihoods of pastoral communities, particularly within arid and semi-arid regions of Africa [[1], [2], [3], [4], [5], [6]]. Because these environments are highly dynamic and support essential functions like livestock production and water-related ecosystem services, consistent monitoring is fundamental for sustainable grazing management and climate-resilient planning [7,8]. However, conventional field-based monitoring is frequently constrained by the vast spatial extent, remoteness, and seasonal variability of these landscapes, which makes repeated ground surveys logistically difficult and expensive [9,10]. While remote sensing and machine learning have improved vegetation mapping, their application in African rangelands is often hindered by data scarcity and environmental complexity [[11], [12], [13]].

The primary motivation behind this methodology is the need to overcome the technical barriers posed by spectral and structural heterogeneity in dryland environments. In these regions, vegetation is often sparse and intermixed with bare soil, dry litter, and biological crusts, creating mixed pixels that reduce the separability of plants from their background [[14], [15], [16]]. This issue is exacerbated in high-resolution drone-based imagery, where individual shrubs appear as complex assemblages of branches, shadows, and senescent material, leading to high-frequency textural noise that challenges traditional supervised classification and threshold-based methods [[17], [18], [19]]. Furthermore, the labor-intensive nature of manual annotation in these complex landscapes limits the creation of the large, reliable datasets required for contemporary machine learning workflows [20,13].

This methodology utilizes generative artificial intelligence and foundation models to address these limitations by reconstructing scenes to isolate ecologically meaningful vegetation while restructuring visually distracting background information [21,22]. By integrating a generative multimodal model with concept-driven segmentation, the methodology is designed to improve vegetation-background separability and facilitate the rapid generation of scalable annotations [23,24]. This approach provides an application-oriented framework for monitoring indigenous forage in data-scarce dryland ecosystems like Marsabit County, Kenya, filling a methodological gap where technical workflows must account for extreme environmental heterogeneity [25,13].

## Method details

### Concept of generative semantic reconstruction

In arid rangelands, optical sensors capture open-canopy mixtures of sunlit leaves, shadowed leaves, soil, and standing dead material [15]. This structural heterogeneity produces high within-scene variability, particularly in the background, where elevated coefficients of variation act as camouflage noise that obscures the target shrub signal.

To address this mixed-pixel problem [26], we employed Generative Domain Adaptation (GDA) to translate data from a visually heterogeneous source domain into an annotation-ready target representation. This image-to-image (*I2I*) translation can be expressed as:

*I_reconstructed_ = G(I_raw_, P)*

where G denotes the generative reconstruction function and P is the semantic prompt used to guide the transformation. The objective was to preserve the original vegetation signal while restructuring visually distracting background information into an annotation-ready representation that maintained plant structure and improved downstream mask generation.

In this study, generative semantic reconstruction is treated as a constrained domain-adaptation step rather than as open-ended image synthesis. The purpose of the reconstruction is to retain the visible morphology and spatial structure of the target vegetation while reducing irrelevant background variability that interferes with annotation. This use of prompt-guided generative reconstruction and foundation-model-assisted segmentation is consistent with recent developments in generative and concept-driven image analysis workflows [21,23,24,22]. The detailed implementation of the staged reconstruction, scene parsing, mask refinement, and final segmentation workflow is described in Section 2.3.

### Dataset description

**Fig. 1:**
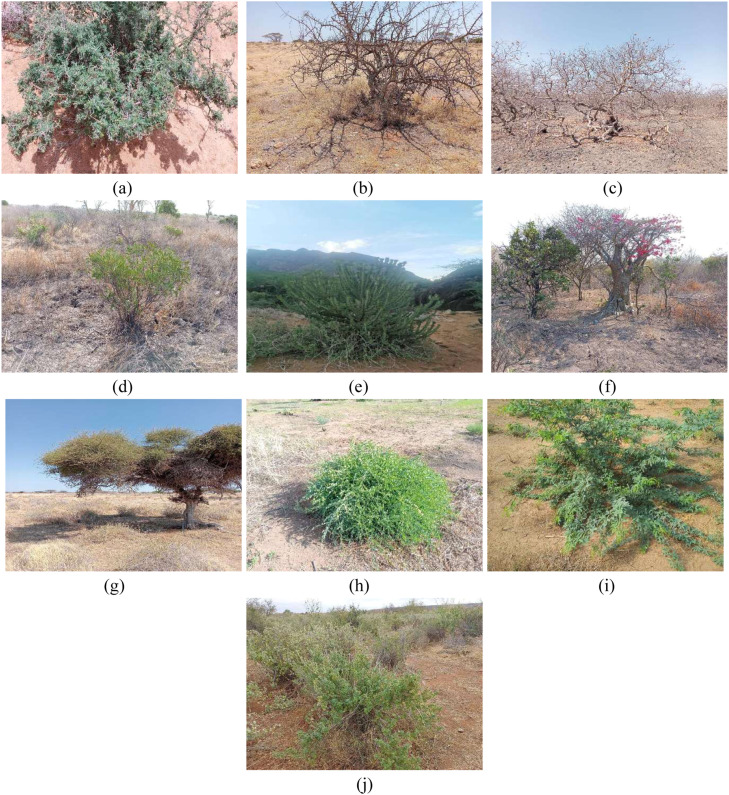
Dataset samples of (a) Acacia (b) Acacia Tree (c) Bissar (d) Boscia Coriacea (e) Candelabra (f) Desert Rose (g) Duma (h) Maerua Edulis (i) Prosopis Juliflora (j) Yabah.

The foundational dataset comprises RGB images collected in the arid and semi-arid lands of Marsabit County, Kenya. Utilizing a participatory approach with local herders, the data captures 10 distinct classes of key indigenous forage species (Fig. 1). A subset of 98 image pairs (Original v/s Generated) was selected to ensure a balanced representation of both structurally simple and complex species for quantitative spectral analysis. Plants were identified and named using local vernacular (Rendille/Borana) and scientifically validated using resources like *African Plants: A Photo Guide* [27,28].

### Architecture

**Fig. 2:**
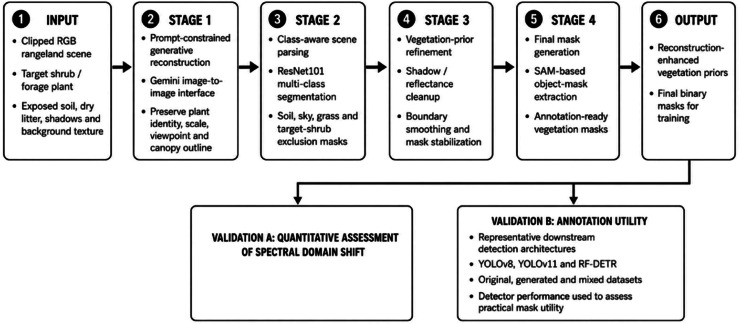
**Overall architecture of the generative semantic reconstruction workflow.** The workflow shows prompt-constrained reconstruction, ResNet101-based scene parsing and exclusion-mask generation, vegetation-prior refinement, SAM-based final object-mask generation, and downstream validation through quantitative spectral-domain assessment and annotation-utility evaluation.

The operational workflow consisted of four sequential stages: generative reconstruction, class-aware scene parsing, refinement of the vegetation prior, and final mask generation (Fig. 2). The scene was first localized, parsed into dominant semantic classes using a ResNet101-based multi-class segmentation module, and reduced to an isolated vegetation foreground through exclusion-mask refinement. Noise suppression and original-signal preservation were balanced across four primary processing stages. Following ResNet101-based parsing, exclusion, and compositional cleanup, the refined reconstruction priors were submitted to SAM 3.0 for final automated object mask generation. In this workflow, SAM 3.0 did not replace the earlier class-aware scene parsing stage; instead, it operated on reconstruction-enhanced vegetation priors to produce final annotation-ready masks. The complete dataset was subsequently partitioned into original, generated, and mixed subsets for quantitative model training and comparative performance assessment.

**Fig. 3:**
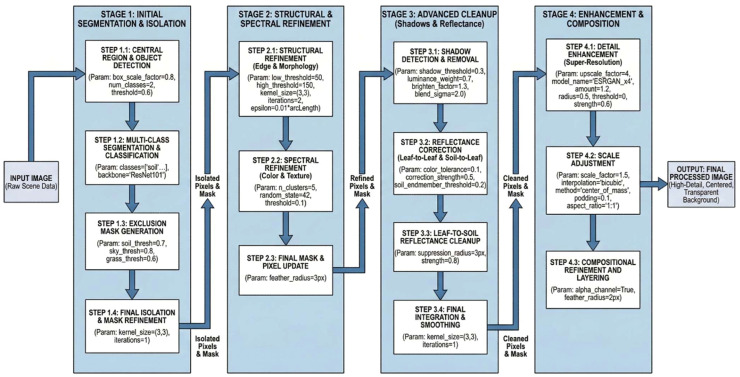
Integrated detailed vegetation segmentation and enhancement pipeline.

The operationalization of the generative reconstruction logic required precise parametric control to balance noise suppression while preserving the original vegetation signal. Computational implementation was therefore defined through explicit hyperparameters across the four primary stages of the workflow (Fig. 3; Table 1). All raw inputs, reconstructed outputs, and segmentation masks were generated and retained at a fixed spatial size of 1184 × 864 pixels. The implementation parameter set reported in Table 1 was treated as authoritative for all experiments. The conceptual prompt diagram is provided only to explain the reasoning structure of the workflow and does not override the implementation defaults reported in the protocol. Parameters listed in Table 1 correspond to the deterministic pre-processing, exclusion-mask refinement, reconstruction cleanup, and output-standardization steps used after generative reconstruction.

**Table 1:** Default implementation parameters used in the generative semantic reconstruction workflow.

**Stage**	**Substep**	**Parameter**	**Default value**
Input	All stages	Canonical image size	1184 × 864 px
1	1.1	box_scale_factor	0.8
1	1.1	num_classes	2
1	1.1	Threshold	0.6
1	1.2	Classes	soil, sky, grass, target_shrub
1	1.2	Backbone	ResNet101
1	1.3	soil_thresh	0.7
1	1.3	sky_thresh	0.8
1	1.3	grass_thresh	0.6
1	1.4	kernel_size	(3,3)
1	1.4	Iterations	1
2	2.1	low_thresh	50
2	2.1	high_thresh	150
2	2.1	kernel_size	(3,3)
2	2.1	Iterations	2
2	2.1	Epsilon	0.01 × arcLength
2	2.2	n_clusters	5
2	2.2	random_state	42
2	2.2	Threshold	0.1
2	2.3	feather_radius	3 px
3	3.1	shadow_threshold	0.3
3	3.1	luminance_weight	0.7
3	3.1	brighten_factor	1.3
3	3.1	blend_sigma	2.0
3	3.2	color_tolerance	0.1
3	3.2	correction_strength	0.5
3	3.2	soil_endmember_threshold	0.2
3	3.3	suppression_radius	3 px
3	3.3	Strength	0.8
3	3.4	kernel_size	(3,3)
3	3.4	Iterations	1
4	4.1	upscale_factor	4
4	4.1	model_name	ESRGAN_x4
4	4.1	Amount	1.2
4	4.1	Radius	0.5
4	4.1	Threshold	0
4	4.1	Strength	0.6
4	4.2	scale_factor	1.5
4	4.2	Interpolation	bicubic
4	4.2	Method	center_of_mass
4	4.2	Padding	0.1
4	4.2	aspect_ratio	1.1
4	4.3	alpha_channel	True
4	4.3	feather_radius	2 px

**Fig. 4:**
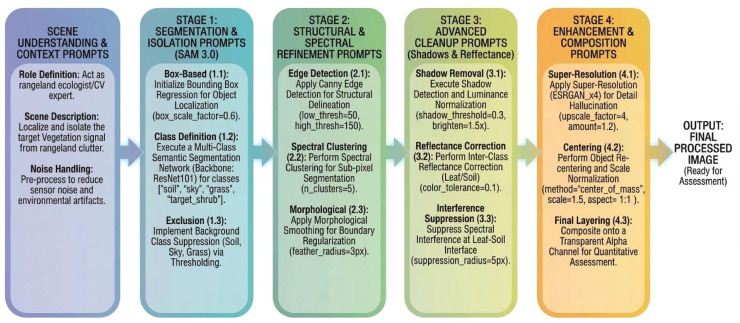
Conceptual prompt logic underlying the generative semantic reconstruction workflow.

This reasoning-driven instruction flow enabled high-fidelity image translation while reducing the risk of hallucinations commonly associated with standard diffusion-based generation workflows [21,24]. Its effectiveness stemmed from the generative semantic reconstruction process, which leveraged structured prompt logic and staged refinement to preserve subject fidelity while suppressing irrelevant background variability. The full instruction flow is summarized in Fig. 4.

#### Generative reconstruction protocol and user-accessible configuration

The generative reconstruction stage was implemented using the image-to-image capability of Gemini Nano Banana Pro accessed through its image-generation interface. The model was accessed through the web-based multimodal generation interface in January 2026. All input images were resized or retained at the canonical spatial resolution of 1184 × 864 pixels and the reconstructed outputs were saved at the same resolution to maintain spatial consistency across the original image, reconstructed image, intermediate segmentation outputs, and final annotation masks.

Since the generative reconstruction model was accessed through a closed multimodal image-to-image interface, low-level diffusion parameters such as sampler type, denoising strength, random seed, CFG scale, and number of diffusion steps were not exposed to the user. To support transparent implementation despite this limitation, all user-accessible configuration elements and reconstruction constraints are reported, including the model interface, access date, image resolution, prompt template, negative constraints, number of reconstruction attempts, output rejection criteria, and deterministic pre- and post-processing parameters used after generation.

For each raw field image, the original image was uploaded as the only visual reference, and the following fixed prompt template was used:

**Prompt used for generative semantic reconstruction:**

“Reconstruct the same target shrub/forage plant as an annotation-ready image. Preserve the original plant identity, branching pattern, leaf distribution, flower and fruit structures, camera viewpoint, object scale, and canopy outline. Suppress the irrelevant background clutter like dry soil, litter, stones, and any visually confusing texture. Do not add new branches, leaves, flowers, stems, fruits, thorns, shadows, or plant parts. Do not remove visible plant organs. Do not stylize the image or generate artifacts. The output should remain a realistic RGB field-image reconstruction suitable for vegetation segmentation and annotation.”

This prompt was intentionally formulated as a constraint-based instruction for maintaining clarity in reconstruction rather than a creative image-generation request. The positive instruction required preservation of plant identity, geometry, viewpoint, object scale, canopy outline, and visible morphological structures. The negative instruction explicitly prohibited hallucination, removal, stylization, or semantic alteration of the target vegetation. The objective was therefore not to generate new plant images but to reconstruct the same scene into an annotation-ready representation while restructuring background spectral and textural interference.

For each image, maximum of 3 reconstruction attempts was made using the same prompt and input image. If the first output satisfied the reconstruction constraints, it was retained. If not, the image was regenerated using the same prompt and constraints in a new session. Generated outputs were rejected if the target plant outline was visibly altered, if new plant organs were hallucinated, if branches, leaves, flowers, fruits, or thorns were removed, if object scale or camera viewpoint changed, or if the background replacement crossed into the target vegetation area. Outputs were also rejected if the generated scene became stylized, cartoon-like, over-smoothed, or visually inconsistent with realistic RGB field imagery.

The final output was selected according to the following rule:

The retained image had to maximize background simplification while preserving the original shrub boundary, internal branching structure, and visible plant organs. Preference was given to the reconstruction that produced the clearest vegetation-background separation without changing the biological form of the plant. This output-selection rule was applied consistently across all species and image pairs.

The controllable and non-controllable parameters of the generative reconstruction stage are summarized in Table 2 below:

**Table 2:** Settings, constraints, and acceptance criteria used for generative background simplification of field images.

**Component**	**Setting used in this study**
Generative model	Gemini image-generation interface, Nano Banana Pro
Access route	Web-based multimodal image-to-image generation interface
Access date/version	January 2026
Input modality	Original RGB field image + text prompt
Input and output image size	1184 × 864 pixels
Prompt type	Fixed constraint-based semantic reconstruction prompt
Negative constraints	No hallucination, no plant-organ addition, no plant-organ removal, no change in viewpoint, no change in scale, no stylization
Number of generations per image	1–3 attempts per image
Output-selection rule	Retain the reconstruction with strongest background simplification and highest preservation of target plant structure
Rejection criteria	Altered canopy outline, hallucinated shrub organs, removed branches/leaves/flowers/fruits/thorns, changed viewpoint or scale, background leakage into vegetation, unrealistic stylization
Random seed	Not accessible
CFG scale	Not accessible
Denoising strength	Not accessible
Sampler	Not accessible
Number of diffusion steps	Not accessible
Temperature	Not accessible
Post-generation processing	Deterministic segmentation and refinement parameters reported in Table 1

This protocol ensured that the generative stage functioned as a semantic reconstruction and background-suppression step, rather than as uncontrolled image synthesis. The retained reconstructed images were subsequently used as vegetation-enhanced priors for the downstream ResNet101-based scene parsing, exclusion-mask refinement, and SAM-based final object-mask generation stages.

#### Computational requirements and scalability

The computational requirements of the workflow were recorded to support practical reuse, implementation transparency, and scalability assessment. The workflow involved three computationally distinct stages: cloud-based generative reconstruction, local or notebook-based segmentation and mask generation, and downstream detector training and inference. The generative reconstruction stage was performed through a closed cloud-based multimodal image-to-image interface. Therefore, backend hardware details, including GPU type, memory, CUDA version, and parallelization strategy, were not visible to the user. For this stage, only the user-observable wall-clock reconstruction time per image could be reported. In contrast, the segmentation, mask-generation, training, and inference stages were executed in the user-controlled computational environment and are therefore reported with hardware and software details.

The computational setup and runtime characteristics of each stage are summarized in Table 3. Values should be interpreted as implementation-specific estimates because runtime depends on image size, internet latency during cloud-based generation, GPU availability, model size, and batch-processing configuration.

**Table 3:** Computational requirements of the generative semantic reconstruction workflow.

**Workflow stage**	**Hardware/software**	**Processing Time**
Generative reconstruction	Cloud-based generative model interface; backend GPU not user-accessible	1–2 min/image wall clock time, including upload, generation, visual inspection and saving
ResNet101 scene parsing	Google Colab CUDA-enabled environment; NVIDIA Tesla T4 GPU, 16 GB VRAM; Python/PyTorch implementation	0.5–2 s/image for 1184 × 864 px images
SAM-based final mask generation	Google Colab CUDA-enabled environment; NVIDIA Tesla T4 GPU, 16 GB VRAM; Intel Xeon CPU; ∼12–13 GB RAM; Python 3.10; PyTorch CUDA implementation; SAM-based automatic mask generation applied to reconstruction-enhanced vegetation priors	2–5 s/image, including image loading, prompt/mask generation, mask selection, post-processing, and export
YOLOv8 training	Google Colab CUDA-enabled environment; NVIDIA Tesla T4 GPU, 16 GB VRAM; Intel Xeon CPU; ∼12–13 GB RAM; Python 3.10; Ultralytics YOLOv8 segmentation implementation with PyTorch/CUDA; image size 640 px; batch size 16; approximately 300 epochs	45–75 min total training time per dataset split
YOLOv11 training	Google Colab CUDA-enabled environment; NVIDIA Tesla T4 GPU, 16 GB VRAM; Intel Xeon CPU; ∼12–13 GB RAM; Python 3.10; Ultralytics YOLOv11/PyTorch implementation; image size 640 px; batch size 16; approximately 300 epochs	50–80 min total training time per dataset split
RF-DETR training	Google Colab CUDA-enabled environment; NVIDIA Tesla T4 GPU, 16 GB VRAM; Intel Xeon CPU; ∼12–13 GB RAM; Python 3.10; PyTorch-based RF-DETR implementation; image size 640 px; batch size 4–8 depending on GPU memory; 42 epochs; best checkpoint selected using validation mAP	20–40 min total training time per dataset split
Inference	Same hardware and software environment as detector training	YOLOv8: ∼0.02–0.05 s/image; YOLOv11: ∼0.03–0.06 s/image; RF-DETR: appx. 0.08–0.15 s/img, including model forward pass and post-processing

### Quantitative assessment metrics

To rigorously evaluate the pipeline, we employed four spectral metrics. These were calculated for both the bush and background regions of every image in the dataset.

**a. Coefficient of Variation:**

$$CoV=\;\left(\frac{\sigma }{\mu }\;*\;100\right)$$

Where σ is the standard deviation and μ is the mean of the pixel intensity values [14]. This metric was used to quantify whether reconstruction altered background variability and target-vegetation texture. It was applied separately to the background (Bg) and target-shrub (bush) masks to distinguish background texture redistribution from changes in vegetation structure.

**b. Shannon Entropy (H'):**

$$H^{\prime }=\;-\sum_{i=1}^{R}p_{i}lnp_{i}$$

Where $p_{i}$ is the proportion of pixels with intensity value *i*, and R is the total number of intensity bins (Convertino et al., 2012). Shannon entropy was used to quantify changes in tonal and textural complexity. In the present workflow, increases in background entropy were interpreted as reconstruction-induced texture redistribution rather than noise removal.

**c. Rao’s Q (Quadratic Entropy):**

$$Q=\;\sum_{i=1}^{R}\sum_{j=1}^{R}d_{ij}p_{i}p_{j}$$

Where $d_{ij}$is the distance between spectral values i and j.

It is a measure of spectral divergence. It accounts for the pairwise distance between pixel intensities. Rao’s Q was used to quantify changes in spectral divergence and to assess whether the reconstruction process increased fine-scale structural differentiation within the target vegetation.

**d. Jeffries-Matusita (JM) Distance:**

$$JM=2\left(1-e^{-B_{ij}}\right)$$

Where $B_{ij}$ is the Bhattacharyya distance between class i (Bush) and class j (Bg) [29]. This is to ensure that the plant is distinct from the background, which is reflected by higher JM. A measure of class separability between the bush and background. It ranges from 0 to 2.0. A value > 1.9 indicates excellent separability. This metric was used to assess whether vegetation-background separability was preserved after reconstruction.

### Downstream model validation

The downstream detector comparison was intended to evaluate annotation utility rather than to serve as a comprehensive benchmark against all recent vegetation segmentation algorithms. To assess the practical utility of the reconstructed data, we trained three representative downstream detection architectures: YOLOv8, a CNN-based detector; YOLOv11, a more recent YOLO-family architecture with enhanced feature extraction capabilities (Ultralytics, 2024); and RF-DETR, a transformer-based detector built on a DINOv2 backbone (Robinson et al., 2025). Model performance was evaluated using mean Average Precision (mAP@50) across three dataset variants: (i) Original Images, (ii) Reconstructed (Generated) Images, and (iii) Mixed Data.

## Method validation

To validate the generative semantic reconstruction pipeline, a paired quantitative assessment was conducted on 98 image pairs across 10 indigenous rangeland species.

### Component-dependency rationale for workflow-level validation

A formal neural-network-style ablation was not performed because the proposed workflow is a sequential annotation-generation protocol rather than an end-to-end trainable architecture. The individual stages are functionally dependent: reconstruction first generates the annotation-ready prior, ResNet101-based parsing provides class-aware exclusion masks, refinement stabilizes the foreground vegetation representation, and SAM-based segmentation is then applied to the refined prior. Removing one stage does not produce a directly comparable variant of the same method, but rather an incomplete workflow with different input assumptions. Therefore, instead of reporting a conventional architectural ablation, we provide a component-role analysis and validate the complete workflow through paired spectral-domain metrics and downstream detector performance. This evaluation is consistent with the objective of the study, which is to present a structured methodology for automated vegetation annotation under spectral heterogeneity.

### Global analysis: spectral-domain shift after reconstruction

**Fig. 5:**
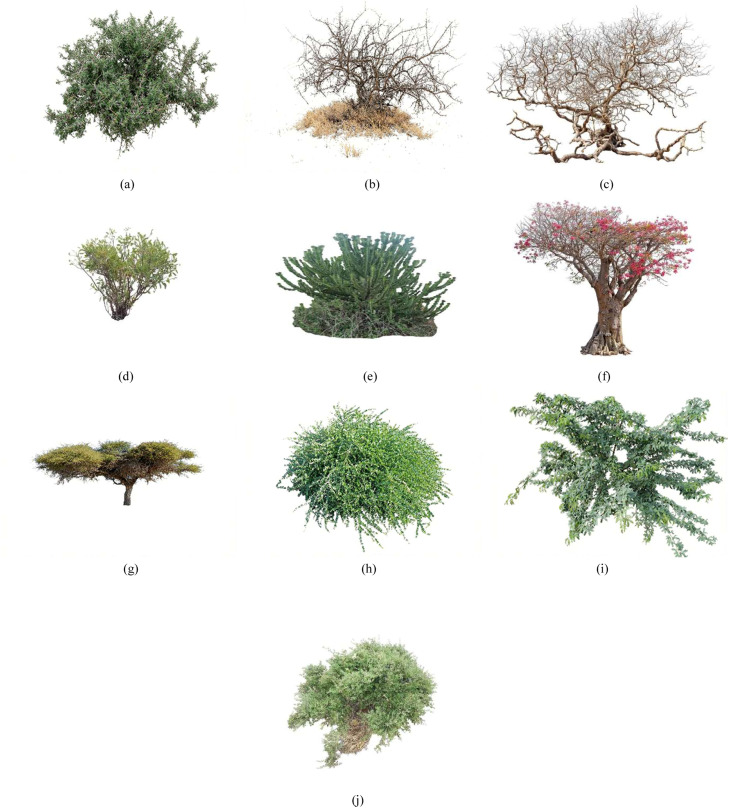
Reconstructed samples of (a) Acacia (b) Acacia Tree (c) Bissar (d) Boscia Coriacea (e) Candelabra (f) Desert Rose (g) Duma (h) Maerua Edulis (i) Prosopis Juliflora (j) Yabah.

**Fig. 6:**
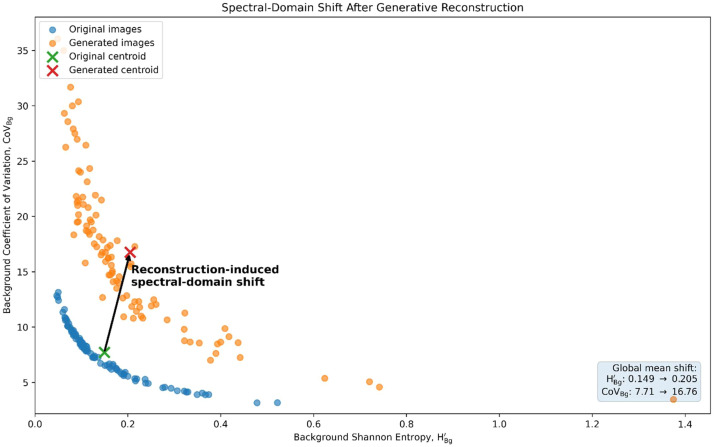
Spectral-domain shift after generative reconstruction.

The paired spectral analysis showed that the generative reconstruction produced a measurable spectral-domain shift rather than a simple clean room reduction in background variance (Fig. 5; Fig. 6; Table 4). The reconstructed images visually reduced distracting background interference and improved the organization of annotation-ready vegetation priors. However, the spectral metrics indicate that this effect is better interpreted as background texture redistribution rather than uniform background noise suppression.

**Table 4:** Species-specific and global spectral domain shift metrics: quantifying post-generative reconstruction.

**Class**	**n**	**Background CoV shift, ΔCoV_Bg_ (%)**	**Texture gain, ΔH’_Bg_ (%)**	**Target-vegetation CoV shift, ΔCoV_Bush_ (%)**	**Separability shift, ΔJM (%)**	**Interpretation**
Acacia	10	+104.6	+26.0	−2.6	−0.3	Background variability increased, while target vegetation texture remained largely stable and JM changed only marginally.
Acacia-Tree	10	+230.5	−27.5	−18.6	+0.1	Strong background CoV increase but reduced entropy and target CoV; JM remained effectively unchanged.
Bissar	10	+131.2	+35.5	+16.4	−0.3	Reconstruction increased both background texture and target-vegetation variability, with only a slight JM decrease.
Boscia-Coriacea	10	+143.6	+31.3	+32.4	−0.4	Increased target structural variability, but background CoV also increased; separability remained near saturation.
Candelabra	10	+44.8	+151.7	+27.7	−3.4	Strong entropy and target-texture gain, but the largest JM reduction among classes, indicating possible foreground-background redistribution.
Desert-Rose	10	+117.1	+67.9	+19.5	−0.4	Background and target texture increased, while class separability changed only slightly.
Duma	8	+93.0	+84.7	+1.9	−1.0	Background and entropy increased; target CoV remained nearly stable, consistent with texture-confusion behaviour in thorny forms.
Maerua-Edulis	10	+121.4	+42.5	+3.9	−0.2	Moderate texture redistribution with minimal change in target variability and separability.
Prosopis-Juliflora	10	+131.9	+46.4	+18.0	−1.8	Increased target and background variability; JM decreased modestly but remained high in absolute terms.
Yabah	10	+129.6	+46.6	+13.6	−0.2	Consistent increase in background and target texture with negligible separability loss.
Global	98	+125.4	+49.8	+11.4	−0.8	Reconstruction produced a spectral-domain shift and moderate target-texture enhancement, but not global background-variance suppression.

**Background CoV shift:** The global mean background coefficient of variation (CoV_Bg_) increased by 125.4 % after reconstruction (Fig. 6; Table 4). Therefore, the increase in CoV_Bg_ suggests that the generative reconstruction replaced the original field background with more structured or redistributed background texture. This is visually useful for annotation.

**Background entropy increase:** Background Shannon entropy (H′_Bg_) increased globally by 49.8 % after reconstruction (Fig. 6; Table 4). This indicates that the generated backgrounds contained more distributed tonal or textural information than the original clipped backgrounds. Rather than an entropy inversion implying simple noise removal, the result is better interpreted as a reconstruction-induced increase in background texture complexity.

**Target-shrub texture preservation:** The coefficient of variation within the target-shrub region (CoV_Bush_) increased moderately by 11.4 % at the global level (Table 4). This suggests that the reconstruction process generally retained, and in some cases enhanced, internal shrub texture and structural variability. This is important for annotation because the goal of the workflow is not to over-smooth the vegetation object, but to preserve the visible morphology of the target shrub while reducing visually confusing background interference.

**Separability stability:** Jeffries-Matusita (JM) separability showed only a small global decrease of −0.8 % after reconstruction (Table 4). This indicates that vegetation-background separability remained close to saturation despite the reconstruction-induced spectral shift. Therefore, the results do not support a claim of improved separability, but they do support the conclusion that class distinction was largely maintained after reconstruction. The workflow therefore preserves vegetation-background separability while restructuring the spectral and textural properties of the scene.

Overall, the updated global analysis demonstrates that the proposed workflow produces a controlled reconstruction-induced domain shift, characterized by increased background CoV, increased background entropy, moderate target-shrub texture enhancement, and largely preserved vegetation-background separability. These results support the use of the method as a reconstruction-assisted annotation protocol.

### Downstream model validation and annotation viability

Recent agricultural computer-vision studies have shown that detector reliability in field imagery is strongly affected by domain shift, spectral regime, image-quality degradation, occlusion, and background variability, and that model assessment should consider robustness and false-detection behaviour rather than relying only on absolute mAP values [30]. Therefore, the downstream validation in the present protocol used YOLOv8, YOLOv11, and RF-DETR as representative architectures to test whether generative semantic reconstruction improves the annotation utility of vegetation priors under visually heterogeneous rangeland conditions.

**Fig. 7:**
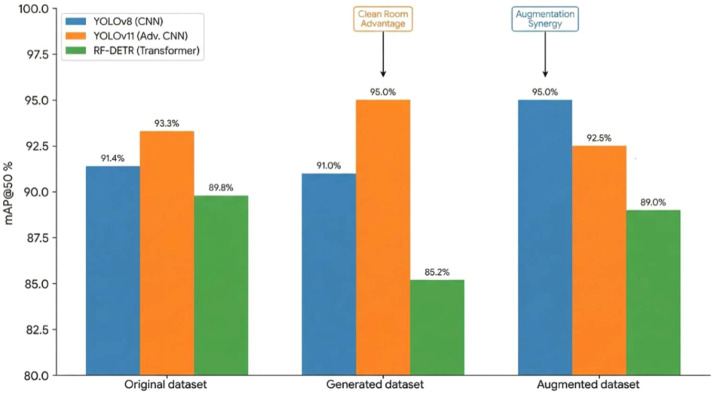
Effect of generative domain shift on detection performance across model architectures.

**CNN Architectures:** Convolutional models exhibited the clearest gains from the reconstructed annotation-ready data. YOLOv11 achieved an increase in mAP@50 from 93.3 % (original) to 95.0 % when trained on generated images, alongside improved recall (86.7 % to 90.4 %). The reconstruction-induced restructuring of visually distracting background texture may have simplified the optimization landscape for the convolutional filters (Fig. 7).

**Transformer Architectures:** The transformer-based RF-DETR model experienced a slight performance drop when trained exclusively on generated data (85.2 % v/s 89.8 % baseline), as transformers rely heavily on global background context to infer scale and presence (Fig. 7). However, training on a mixed dataset recovered performance (90 %) while significantly reducing FPs in soil-dominated regions.

**Automated Annotation:** Segmentation masks produced by the combined ResNet101-to-SAM 3.0 annotation pipeline on reconstructed images were retained without additional correction when they satisfied the predefined reconstruction and mask-quality criteria, and they consistently supported competitive detection performance across all tested architectures.

### Environmental-condition-wise robustness assessment

To avoid overstating geographic generalizability, the available Marsabit image pairs were additionally examined according to visible environmental and structural conditions, including sandy soil, rocky/lithic substrate, dry litter, shadowed scenes, sparse canopy, dense/thorny canopy, and structurally complex shrubs. Qualitatively, the workflow showed the clearest reconstruction benefit in sandy and visually homogeneous dry-soil backgrounds, where vegetation-background separation appeared most stable. Rocky and litter-dominated backgrounds also benefited from background simplification, although residual confusion remained where dry litter or lithic texture resembled woody shrub components. Dense and thorny canopies and strongly shadowed scenes represented the most difficult cases because foreground structure and background texture were visually similar in RGB imagery. These observations support robustness across the tested Marsabit rangeland conditions but do not establish broader geographic generalizability, which should be evaluated using independent dryland datasets.

## Method limitations

The principal methodological limitation of the present workflow is the texture confusion boundary, prominently observed in texturally complex species like *Duma* (*Commiphora* spp.). When a plant's thorny architecture statistically mimics the high-frequency noise of the lithic background, RGB-only generative models struggle to separate the two without depth cues. The spectral analysis further shows that reconstruction does not necessarily reduce background variance. Instead, it may increase background CoV while preserving near-saturated JM separability. Finally, while CNNs excel with this data, transformer architectures like RF-DETR require a mixed dataset containing original images; generative reconstruction obscures the global contextual cues that their attention mechanisms rely on. Future work must focus on integrating depth channels to fully resolve complex plant morphologies [31].

## Related research article

None.

## Declaration on the use of AI in the writing process

During the preparation of this manuscript, the authors used ChatGPT to creatively enrich the graphical abstract and summarize specific text sections. Following the use of these tools, the authors thoroughly reviewed, edited, and validated the content. The authors take full responsibility for the integrity and final content of the manuscript.

## Declaration of competing interest

The authors declare that they have no known competing financial interests or personal relationships that could have appeared to influence the work reported in this paper.

## Data Availability

Data will be made available on request.
